# Deep Dermal and Subcutaneous Deposits in Thin Melanoma: A Cautionary Tale

**DOI:** 10.1111/cup.70025

**Published:** 2025-11-27

**Authors:** Angela Cheng, Jennette Gruchy, Ariel Burns, Richard Langley, Jason Williams, Ryan DeCoste

**Affiliations:** ^1^ Department of Pathology Dalhousie University Halifax Nova Scotia Canada; ^2^ Division of Dermatology, Department of Medicine Dalhousie University Halifax Nova Scotia Canada; ^3^ Division of Plastic Surgery, Department of Surgery Dalhousie University Halifax Nova Scotia Canada

**Keywords:** deposits, melanoma, microsatellites, microsatellitosis, thin melanoma

## Abstract

Melanoma microsatellites are peritumoral metastatic deposits and surrogates for potentially aggressive biological behavior. Their presence indicates clinical stage III disease. They are rarely reported in association with thin primary tumors (< 1.0 mm, pT1). As a poor prognostic factor, the identification of microsatellitosis in an otherwise localized, thin primary melanoma would result in upstaging and additional investigative and therapeutic considerations. Therefore, when microsatellitosis is suspected, efforts should be made to exclude possible mimics. We present three cases of thin melanomas with deep dermal/subcutaneous deposits to highlight the importance of careful and thorough gross and microscopic examination of all melanoma cases, regardless of T‐category.

**Abbreviations**
AJCC: American joint committee on cancer
DFS: disease‐free survival
H&E: hematoxylin and eosin
HMB45: human melanoma black 45
IHC: immunohistochemistry
MART1: melanoma antigen recognized by T‐cells 1
MIS: melanoma in situ
MITF: microphthalmia‐associated transcription factor
MSS: melanoma‐specific survival
OS: overall survival
SLNB: sentinel lymph node biopsy
SOX10: SRY‐related HMG box gene 10

## 1 Introduction

Cutaneous melanoma is the fifth most diagnosed malignancy when squamous and basal cell carcinomas are excluded. It is estimated that in 2025, there will be 104 960 new cutaneous melanoma cases and 8430 related deaths in the United States [1]. In general, thin, localized disease can be excised with excellent prognosis, whereas advanced disease often requires adjuvant systemic therapy and is associated with worse outcomes.

As per the 8th Edition of the American Joint Committee on Cancer (AJCC) Staging Manual, pathologic staging of melanoma is dependent on Breslow thickness and ulceration (pT), regional lymph node metastasis (pN), and distant metastasis (M) [2]. The N‐category also includes in‐transit, satellite, and microsatellite metastases, which are non‐nodal, locoregional deposits thought to derive from intra‐lymphatic spread. Clinically evident deposits are classified as satellites if within 2 cm of the primary tumor or in‐transit metastases when further. Microsatellites are appreciated only by microscopy, without size or distance criteria [2]. These deposits are associated with unfavorable outcomes, usually presenting with thick primary tumors and nodal spread [3]. Locoregional, non‐nodal metastases may also influence the sentinel lymph node biopsy (SLNB) consideration.

Herein, we describe three cases involving thin melanomas with microsatellitosis. Recognizing the significant implications of regional spread in cutaneous melanoma, diagnostic accuracy and quality are paramount. Our cases serve as a teaching and discussion opportunity regarding specimen collection, tissue processing, and histopathologic assessment of all melanocytic lesions, regardless of thickness. To our knowledge, this is the first report focusing on microsatellitosis in thin melanoma.

## 2 Case 1

A 61‐year‐old female with no personal or family history of melanoma presented with a 1‐year history of a right thigh lesion and adjacent dermal nodule. Physical examination revealed a 0.5 cm atypical, pigmented lesion with a discrete subcutaneous induration giving the impression of a possible scar.

**FIGURE 1:**
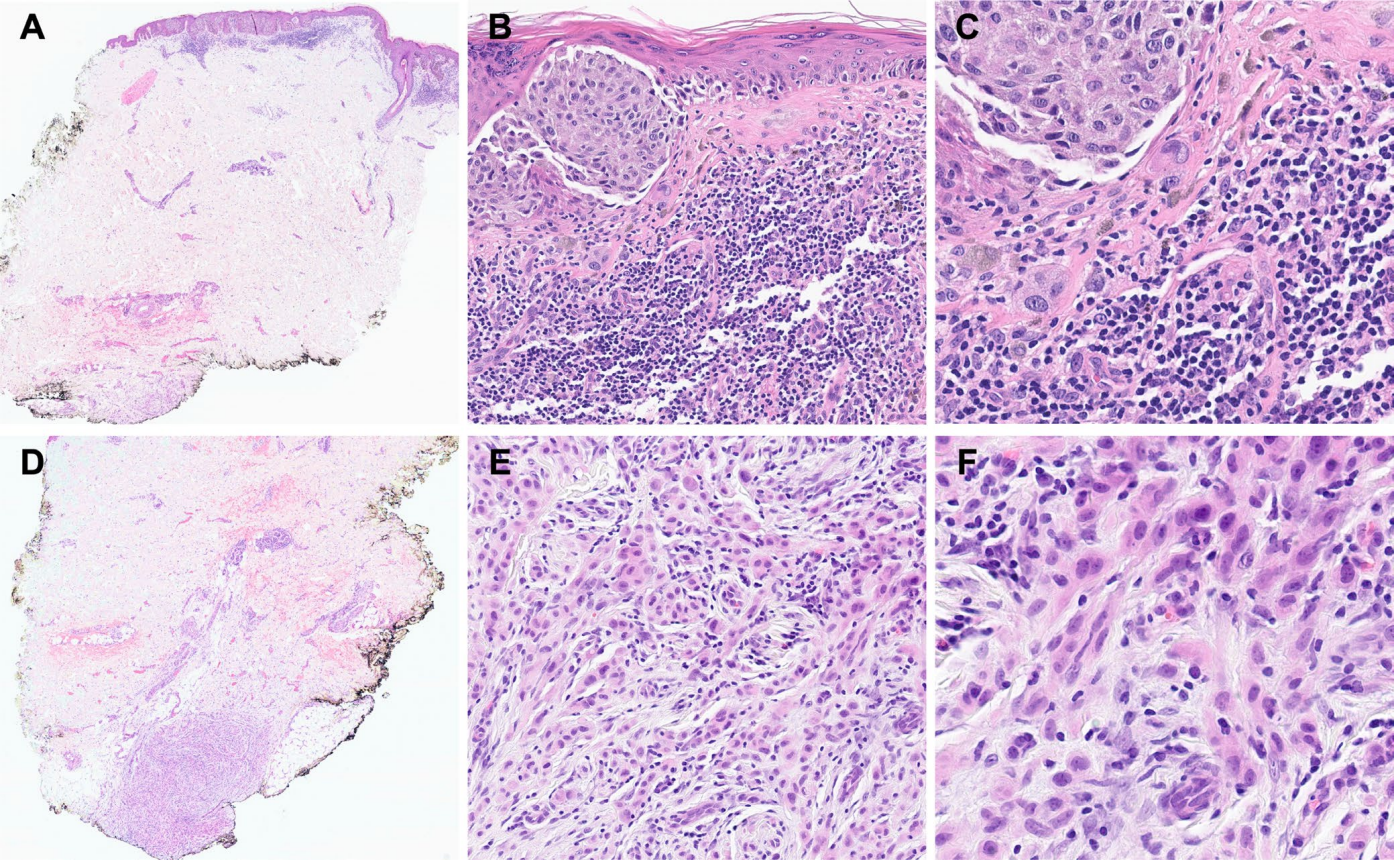
Histopathology of the biopsy specimen from the right thigh of a 61‐year‐old female. (A) A thin primary tumor with a discrete, distant, deep dermal deposit. H&E, 20× magnification. (B) Marked inflammation, fibrosis, and melanophages, features of regression, are associated with the primary tumor. H&E, 200× magnification. (C) The primary tumor consists of nests of highly atypical epithelioid cells. H&E, 400× magnification. (D) The deep dermal deposit extends to the deep margin. H&E, 20× magnification. (E) The deposit with associated inflammation. H&E, 100× magnification. (F) The deposit consists of highly atypical epithelioid cells, like the overlying tumor. H&E, 200× magnification.

**FIGURE 2:**
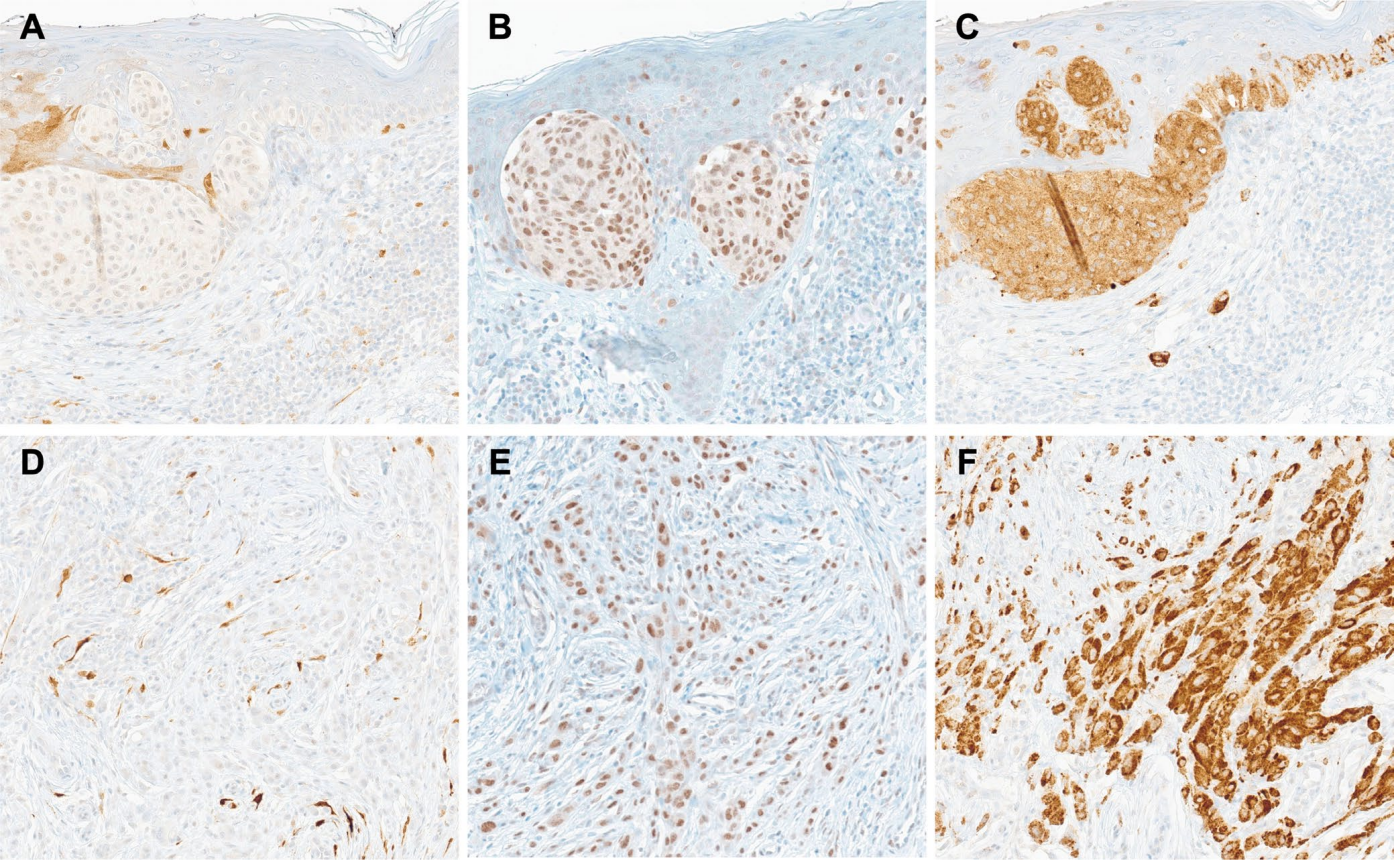
Immunohistochemistry of the biopsy specimen from the right thigh of a 61‐year‐old female, 200× magnification (A) Primary tumor, P16 (B) Primary tumor, PRAME (C) Primary tumor, *BRAF* V600E (D) Dermal deposit, P16 (E) Dermal deposit, PRAME (F) Dermal deposit, *BRAF* V600E.

The biopsy specimen contained a thin melanoma with features of regression, including lymphocytic infiltrates, fibrosis, and melanophages (Table 1). A separate, subcutaneous deposit of atypical, mitotically active epithelioid cells was present, reaching the deep margin; this was not identified grossly (Figure 1). Immunohistochemistry showed SOX10, S100, PRAME, *BRAF* V600E, and elevated Ki‐67 expression in both lesions, and negative p16 (Figure 2). MART1 and HMB45 were expressed by the primary tumor only. Additional levels showed no existing or regressed connection between the lesions, nor any nearby adnexal structures involved by melanoma in situ (MIS). This was classified as a (micro)satellite (N1c).

**TABLE 1:** Clinicopathologic features of cases.

Case	Age	Sex	Location	Breslow depth (mm)	Ulceration	Pathologic stage	Molecular
1	61	F	Right thigh	0.5	No	pT1a	*BRAF* V600E
2	66	F	Right elbow	0.2	No	pT1a	Not performed*
3	77	M	Left parietal scalp	0.7	No	pT1a	*BRAF* V600K

* The patient did not wish to pursue systemic therapy, and therefore, molecular studies were not performed.

SLNB showed no nodal involvement. The patient received 1 year of adjuvant systemic therapy (initially dabrafenib and trametinib, switched to pembrolizumab for toxicity) with no clinical recurrence at 1.5‐year follow‐up.

## 3 Case 2

A 66‐year‐old female with no personal or family history of melanoma presented with a 1‐year history of an evolving pigmented right elbow lesion. On examination, the lesion measured 1.2 cm and appeared highly suspicious for melanoma.

**FIGURE 3:**
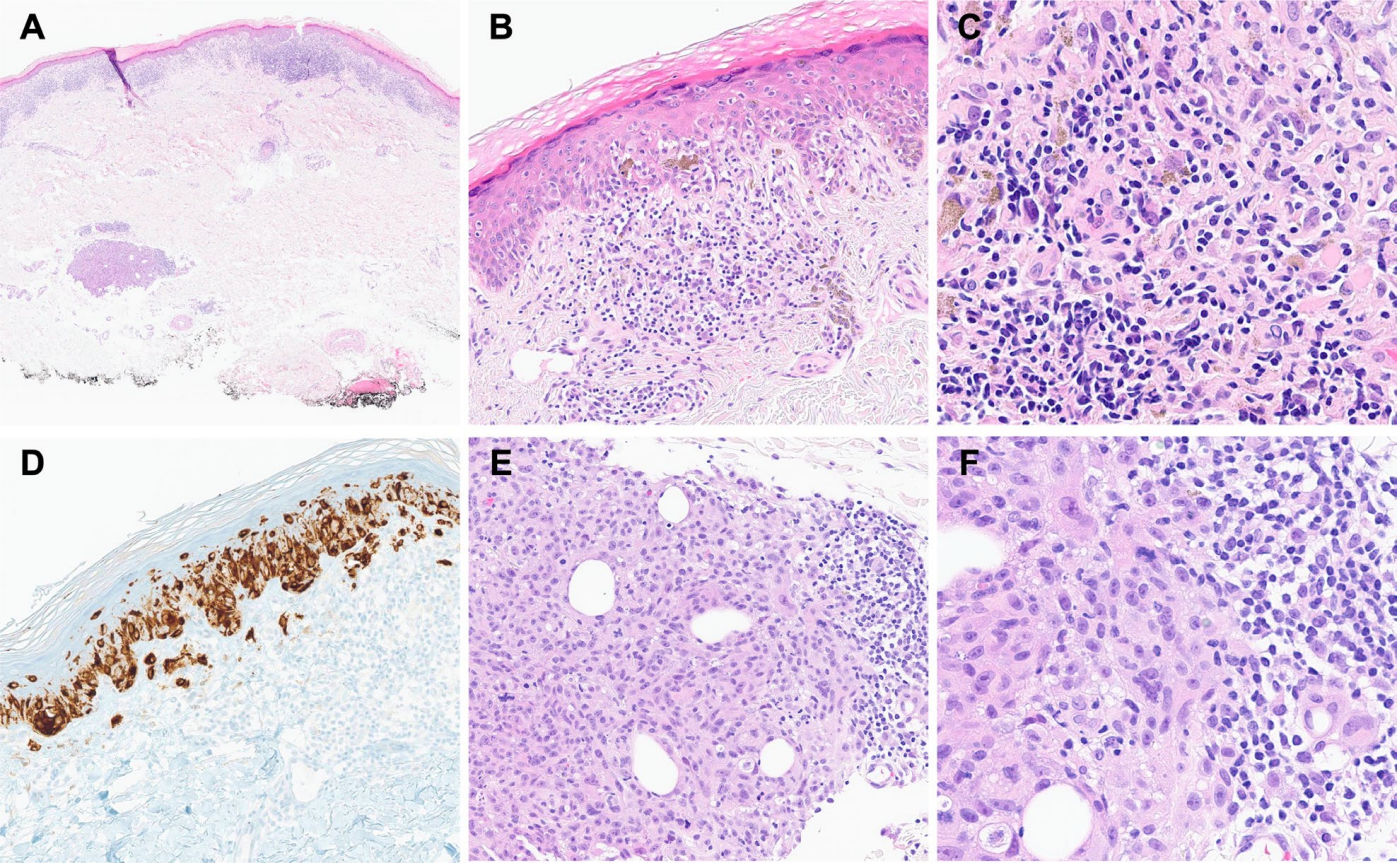
Histopathology of the biopsy specimen from the elbow of a 66‐year‐old female. (A) A thin primary melanoma with a discrete microsatellite. H&E, 20× magnification. (B) Features of regression, including chronic inflammation, fibrosis, and melanophages, are associated with the primary tumor. H&E, 200× magnification. (C) Superficial regression, H&E, 400× magnification. (D) The primary tumor shows expression of MART1 detected by immunohistochemistry 200×. (E) The microsatellite consists of atypical, mitotically active cells, with a rim of associated chronic inflammation. H&E, 200×. (F) The microsatellite is composed of highly atypical, pleomorphic cells, H&E, 400×.

Biopsy showed a thin melanoma with regression (Table 1). The tumor showed MART1, MITF, PRAME, and elevated Ki‐67 expression, and negative p16, by IHC. A separate deposit of atypical, poorly differentiated malignant cells with a brisk lymphocytic inflammatory response was in the deep dermis; this was not recognized grossly (Figure 3). There was no existing or regressed connection between the lesions, nor any nearby adnexal MIS. The deposit was present only in a single hematoxylin and eosin (H&E) stained section, preventing further classification by IHC. Based on the histomorphology and overall scenario, the deposit was classified as a microsatellite (N1c). Clear margins were achieved with re‐excision, and SLNB was negative for metastatic melanoma. The patient chose close radiologic surveillance over adjuvant systemic therapy, with no clinical recurrence at 1.5‐year follow‐up.

## 4 Case 3

**FIGURE 4:**
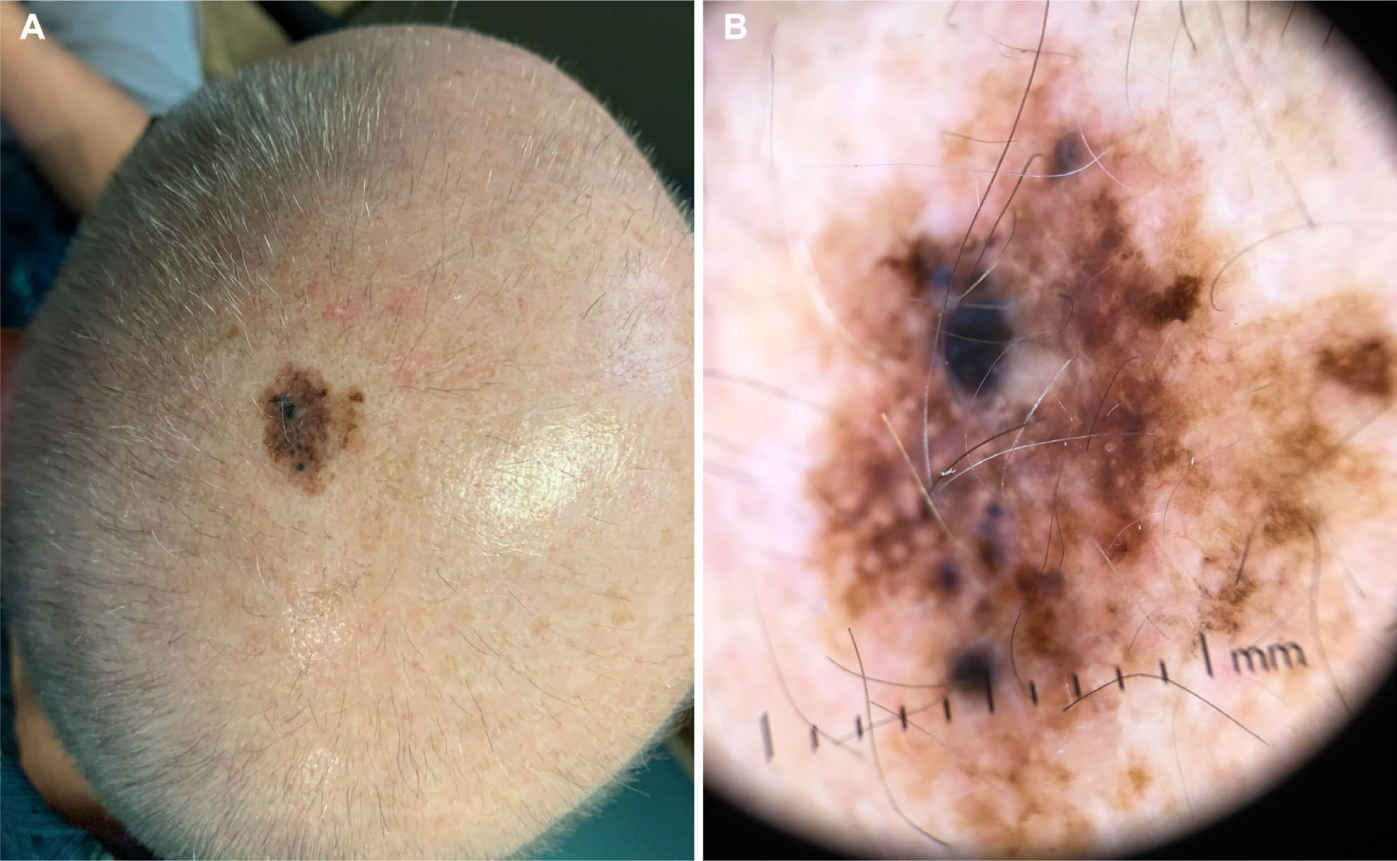
Scalp lesion of a 77‐year‐old male. (A) Clinical photograph showing a highly atypical, pigmented lesion of the scalp. (B) Dermoscopy image of the scalp lesion.

A 77‐year‐old male with a personal history of asynchronous MIS (abdomen, chest) presented with a 2‐year history of an evolving left parietal scalp lesion. Cutaneous examination revealed a highly atypical, 2.1 cm pigmented lesion (Figure 4).

**FIGURE 5:**
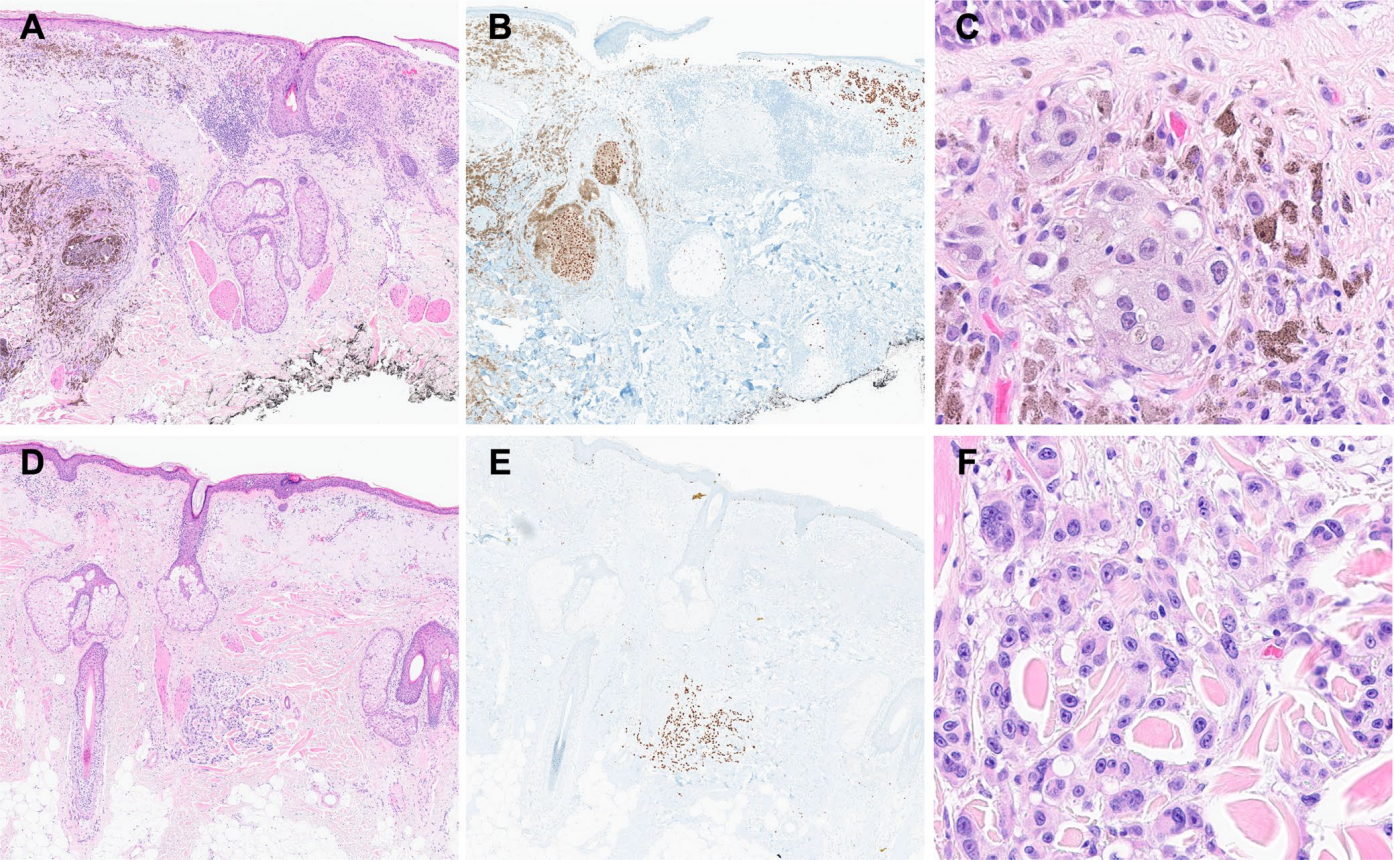
Histopathology of the biopsy and excision specimens from the scalp of a 77‐year‐old male. (A, B) The biopsy specimen shows a thin primary melanoma and marked MIS extending along an adnexal structure, but no additional lesions. H&E (A), SOX10 (B), 40× magnification. (C) The primary melanoma is composed of nests of highly atypical, epithelioid cells with associated lymphocytic infiltrate, melanophagocytosis, increased dermal vascularity, and fibrosis. H&E, 400× magnification. (D, E) The excisional specimen contains a microsatellite. H&E (D), SOX10 (E), 40× magnification. (F) The microsatellite is composed of highly atypical cells. H&E, 400× magnification.

Biopsy showed a thin melanoma with adnexal extension of MIS. The definitive excision specimen contained residual melanoma and a distant, separate, deep dermal deposit with no adnexal MIS or scar (Table 1). There was primary tumor regression. Both lesions expressed MART1 and SOX10 (Figure 5), but not *BRAF* V600E. The primary tumor showed PRAME and elevated Ki‐67 expression, and negative p16, while the deposit was lost on these sections. The deposit was classified as a microsatellite (N1c). Next‐generation (NGS) sequencing identified a *BRAF* V600K mutation. Three months after the excision, the patient developed metastatic melanoma to cervical nodes. The patient was started on pembrolizumab but transitioned to ipilimumab and nivolumab after developing brain metastases, and finally, to encorafenib and binimetinib after developing bone and liver metastases, but unfortunately, deceased.

## 5 Discussion

Microsatellitosis in melanoma is a poor prognostic factor and conveys clinical stage III disease. It is rarely reported in association with a thin primary tumor. We report three cases that demonstrate this phenomenon.

Our first case demonstrated the expression of melanocytic markers (S100, SOX10) and *BRAF* V600E mutant protein by both lesions, supporting melanocytic origin and confirming clonal relationship, respectively. Notably, *BRAF* is the most frequently mutated oncogene in cutaneous melanoma [4]. The absence of HMB45 and MART1 expression in the deposit is likely due to the known immunophenotypic heterogeneity of melanoma; this has been particularly demonstrated with cytoplasmic markers targeting melanosome components in large primary or metastatic lesions [5].

Our second case contained a small deposit, which was absent on additional sections, precluding immunohistochemical assessment. Here, the presumptive diagnosis of microsatellitosis was based on proximity to the primary melanoma, as well as shared histomorphology and inflammatory host response between the deposit and overlying tumor. As a cautious approach, the unlikely possibility of a superficial soft tissue metastasis from another origin was discussed with clinicians; ultimately, a microsatellite deposit of melanoma was deemed, in all probability, the most appropriate diagnosis.

In our final case, microsatellitosis was identified only on the excision specimen. The diagnosis was supported by the expression of melanocytic markers (MART1, SOX10). NGS identified a *BRAF* V600K mutation.

Microsatellitosis in thin melanoma is rarely discussed in the literature but can have significant consequences if missed in practice, particularly with a thin primary given the prognostic disparities. In microsatellite‐focused case–control studies, 1.9%–9.2% of cases occurred with thin primary disease [3, 6, 7, 8]. However, the prevalence of microsatellitosis among thin melanoma cohorts has not been rigorously studied, to our knowledge. Studies involving all comers with thin melanoma have shown excellent prognosis in these cases, including a 12‐year overall survival (OS) of 85.3%, and 10‐, 20‐, and 30‐year melanoma‐specific survivals (MSS) of 97%, 95%, and 94.9%, respectively [9, 10, 11]. In contrast, microsatellitosis has been shown to be an independent risk factor for worse disease‐free survival (DFS), OS, and MSS [3, 6]. Our last case highlights this potential for aggressive behavior, even within a short follow‐up interval. The remaining cases will be subject to close long‐term follow‐up.

The rate of SLNB positivity in all comers with thin melanoma is low, reported as 5.2%; however, certain features confer higher risk, including ulceration, ≥ 0.75 mm Breslow depth, and microsatellitosis [12]. Furthermore, microsatellitosis is reported as an independent predictor for SLN positivity [3]. As such, the NCCN Clinical Practice Guidelines in Oncology (NCCN Guidelines) suggest at least the consideration of SLNB in all patients with microsatellites, regardless of thickness [13]. Conversely, usually only T1b (0.8–1.0 mm or ulcerated) thin melanomas are offered SLNB in the absence of microsatellites [13, 14]. Further, patients with microsatellites may be considered for possible adjuvant systemic (targeted therapy or checkpoint inhibitor immunotherapy) or locoregional therapy (interleukin‐2 intralesional injection) [13, 15].

The identification of microsatellitosis has significant implications, particularly with thin primary disease. We noted features of regression in all our cases, including lymphocytic infiltrates, dermal fibrosis, melanophagocytosis, and telangiectasia. Suggesting these primary tumors may once have been thicker. Regression may be a risk factor for microsatellitosis in pT1 lesions, an area for future studies. The authors suggest attention to and reporting of regression in thin melanoma cases for this reason. Care must be taken when a deposit is recognized to confirm the diagnosis and rule out potential mimickers, such as irregular lobulated extensions of the primary tumor (including adnexal involvement), discontinuity following primary tumor regression, metastases of other origin, and contaminants. Review of clinical history, assessment of level sections, use of ancillary tests, consultation with colleagues, and laboratory quality control are all valuable.

Ultimately, our report draws attention to the rare but significant phenomenon of microsatellitosis in thin primary melanoma. Missing a dermal deposit, particularly in a thin primary tumor, has significant consequences. Thorough histopathological examination is crucial regardless of T‐category.

## Author Contributions

**Angela Cheng:** manuscript writing/editing, figure design; **Jennette Gruchy** and **Ryan DeCoste:** idea conception, manuscript writing/editing; **Ariel Burns** and **Jason Williams:** patient consenting, manuscript writing/editing; **Richard Langley:** patient consenting, manuscript writing/editing, clinical photography.

## Ethics Statement

The authors have nothing to report.

## Conflicts of Interest

The authors declare no conflicts of interest.

## Data Availability

Data sharing not applicable to this article as no datasets were generated or analysed during the current study.
